# Bioanalytical technologies using temperature-responsive polymers

**DOI:** 10.1007/s44211-024-00545-3

**Published:** 2024-04-07

**Authors:** Kenichi Nagase

**Affiliations:** 1 Graduate School of Biomedical and Health Sciences, Hiroshima University, 1-2-3 Kasumi, Minami-ku, Hiroshima, 734-8553 Japan; 2 Faculty of Pharmacy, Keio University, 1-5-30 Shibakoen, Minato, Tokyo, 105-8512 Japan

**Keywords:** Temperature-responsive chromatography, Thermoresponsive polymers, Bioseparation, Biopharmaceuticals, Therapeutic drug monitoring

## Abstract

In recent decades, various bioanalytical technologies have been investigated for appropriate medical treatment and effective therapy. Temperature-responsive chromatography is a promising bioanalytical technology owing to its functional properties. Temperature-responsive chromatography uses a poly(*N*-isopropylacrylamide)(PNIPAAm) modified stationary phase as the column packing material. The hydrophobic interactions between PNIPAAm and the analyte could be modulated by changing the column temperature because of the temperature-responsive hydrophobicity of PNIPAAm. Thus, the chromatography system does not require organic solvents in the mobile phase, making it suitable for therapeutic drug monitoring in medical settings such as hospitals. This review summarizes recent developments in temperature-responsive chromatography systems for therapeutic drug monitoring applications. In addition, separation methods for antibody drugs using PNIPAAm are also summarized because these methods apply to the therapeutic drug monitoring of biopharmaceutics. The temperature-responsive chromatography systems can also be utilized for clinical diagnosis, as they can assess multiple medicines simultaneously. This highlights the significant potential of temperature-responsive chromatography in medicine and healthcare.

**Graphical abstract:**

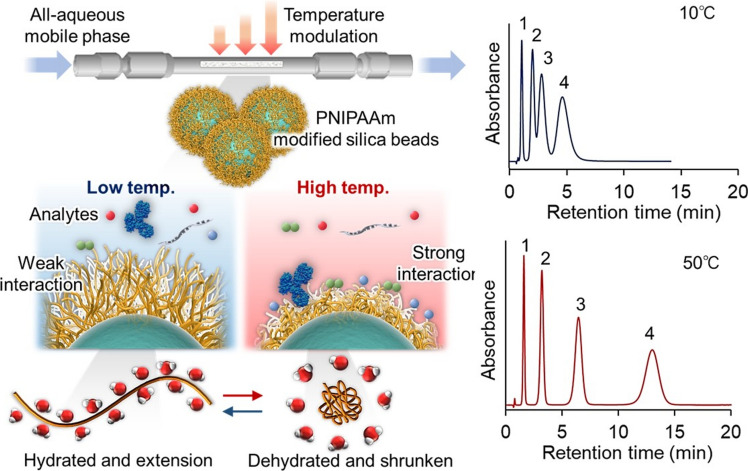

## Introduction

In recent decades, biomedical analysis has increased in importance in the clinical field because analyzing drugs, biomarkers, and bioactive concentrations in the living body is important for appropriate medical treatment and effective therapy [1–5]. Therapeutic drug monitoring (TDM) is essential for biomedical analysis. TDM is the method for measuring drug concentration in serum and is an effective approach to monitor the therapeutic effect using specific types of drugs [6, 7]. The appropriate dosage of certain medications must be modified by assessing the concentration of the drug in the blood serum, as an insufficient concentration of the drug will prove ineffective, whereas an excessively high concentration can lead to toxic side effects. Thus, TDM is required to monitor the drug concentration.

**Fig. 1:**
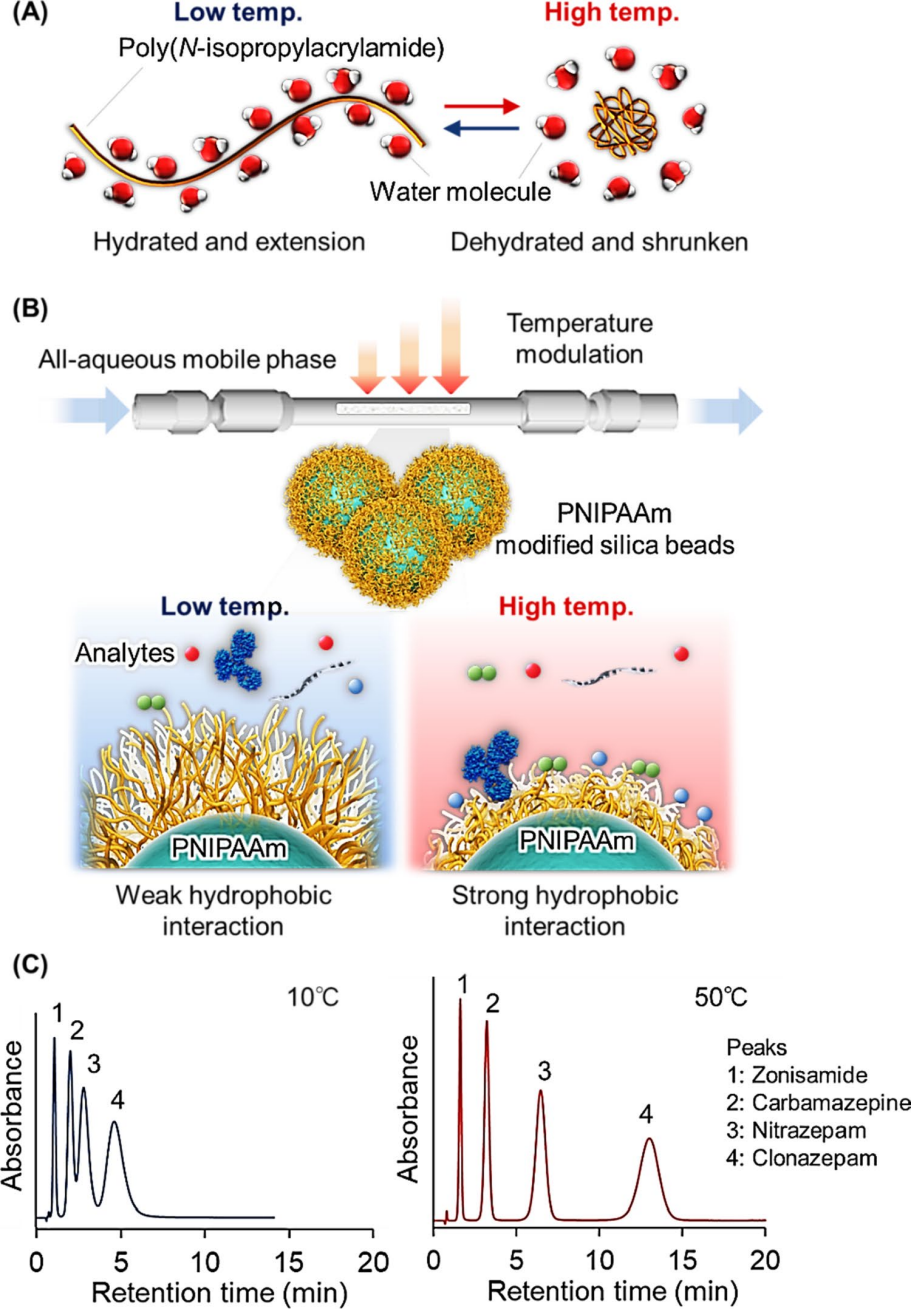
Thermoresponsive polymer poly(*N*-isopropylacrylamide) and its application in temperature-responsive chromatography. (**A**) Thermoresponsive polymer PNIPAAm properties, (**B**) Concept of temperature responsive chromatography, and (**C**) Chromatogram of the drugs at 10 and 50 °C

Liquid chromatography (LC) is a reliable method for measuring drug concentration in serum because of its adaptability and the absence of a specific antibody for each individual drug [8]. However, most of LC requires the presence of organic solvents in the mobile phase to regulate drug retention in the chromatography column. Furthermore, it is necessary to deproteinate the blood samples using organic solvents before determining the drug concentration in the serum using chromatography. Organic solvents are commonly prohibited in hospitals owing to their potential hazards. Therefore, a technique that does not require organic solvents is desirable for TDM. Chromatography, which utilizes poly(*N*-isopropylacrylamide) (PNIPAAm) to respond to temperature changes is a measurement technique, and it does not employ organic solvents in mobile phase [9–29] (Fig. 1).

PNIPAAm is a thermoresponsive polymer whose hydrophobicity changes in response to temperature changes (Fig. 1A). The hydrophilic and hydrophobic properties of PNIPAAm, which are influenced by temperature, change as a result of hydration and dehydration at a lower critical solution temperature (LCST) of 32 °C [30–36]. Also, the PNIPAAm exhibits temperature-modulated extension and shrinking attributed to the hydration and dehydration. This thermoresponsive polymer has been widely utilized in biomedical applications because of its relatively low LCST, which is close to the human body temperature. Examples of these applications include temperature-controlled drug and gene delivery systems [37–44], biosensors and bioimaging systems [45–52], nanoactutors [53–59], bioseparation tools [60–74], cell separation materials [75–90], and cell culture substrates for tissue engineering [91–110].

The ability of PNIPAAm to change its properties in response to temperature has been harnessed for the development of bioanalysis systems such as temperature-responsive chromatography (Fig. 1B). In this technique, the packing beads of the chromatography columns were modified with PNIPAAm, and the interactions between the material and target drugs were adjusted by altering the column temperature.

In an ordinary chromatography system using octadecylsilyl (ODS) group-modified bead-packed columns, hydrophobic interactions between analytes and ODS groups are modulated by the addition of an organic solvent to the mobile phase. By contrast, the PNIPAAm-modified stationary phases were used in temperature-responsive chromatography (Fig. 1B). Because the hydrophobic properties of PNIPAAm can be influenced by external temperature changes owing to hydration and dehydration of the polymer, the surface properties of the PNIPAAm-modified stationary phase can be easily adjusted by altering the column temperature. This allows for the modulation of the hydrophobic interactions between the analytes and the stationary phase. Therefore, retention modulation is quite simple compared to the adjustment of the mobile phase composition (Fig. 1C). The chromatographic system does not require the addition of an organic solvent to the mobile phase and allows for analysis using an all-aqueous mobile phase. Chromatography systems prevent exposure to organic solvents in hospitals, making them suitable for TDM.

This review describes recent developments in TDM techniques using PNIPAAm and their properties. In addition, separation methods for antibody drugs using PNIPAAm, which are applicable for TDM, are also summarized.

## TDM using PNIPAAm modified beads packed chromatography column

HPLC columns using PNIPAAm-modified beads have been developed as innovative chromatography methods because the chromatography system does not require the addition of an organic solvent to the mobile phase, and analyte retention can be modulated by simply changing the column temperature. Therefore, chromatography columns have been investigated for applications in TDM because an all-aqueous mobile phase (without organic solvents) is suitable for use in medical settings.

**Fig. 2:**
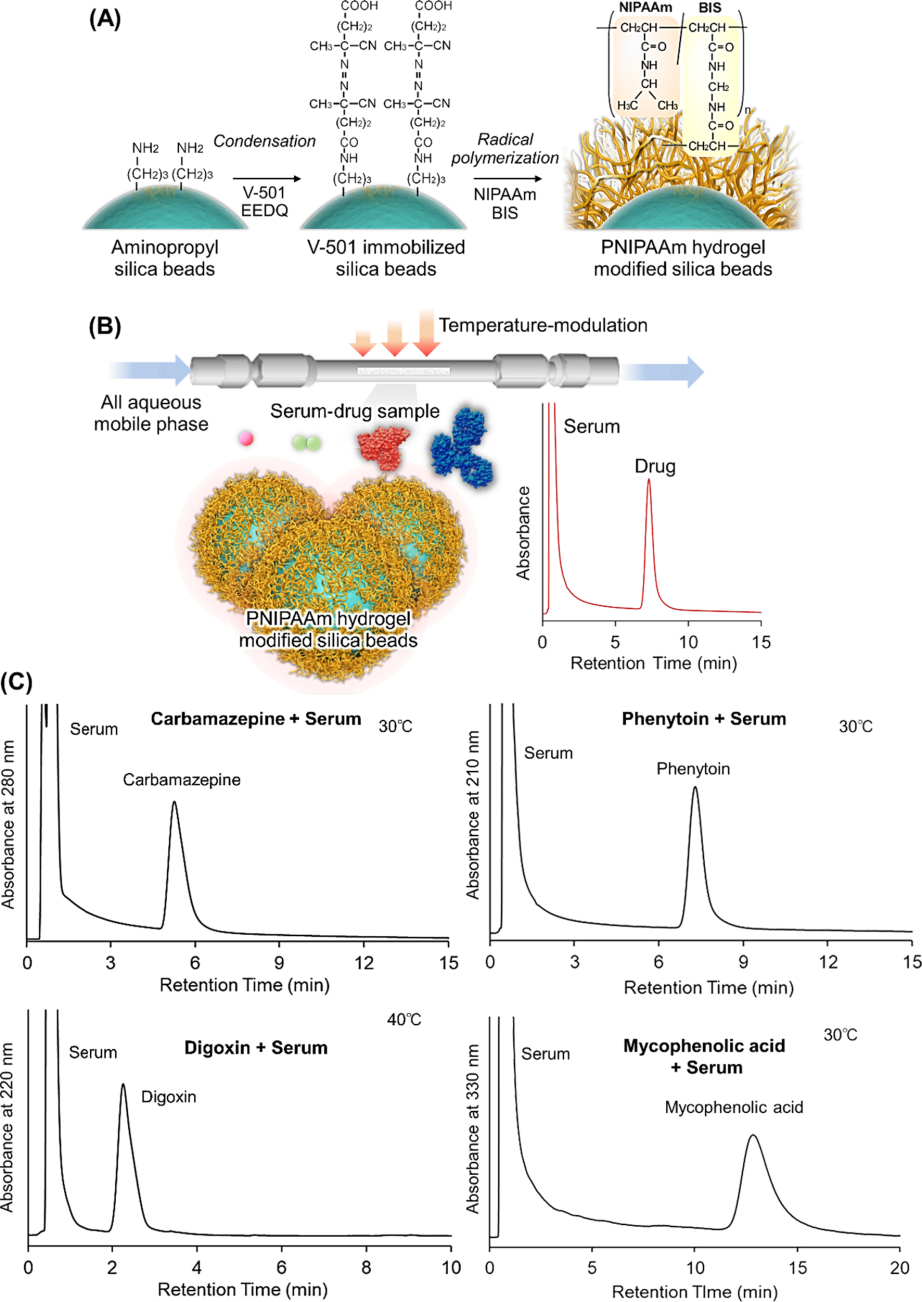
Temperature-responsive chromatography for TDM. (**A**) Preparation scheme for PNIPAAm hydrogel-modified silica beads, (**B**) Conceptual diagram of TDM using temperature-responsive chromatography, and (**C**) Chromatograms of the drug samples with the serum

A temperature-responsive chromatography column with thin PNIPAAm hydrogel layer-modified silica beads was used for TDM (Fig. 2) [111]. PNIPAAm hydrogel modification was performed using the radical initiator V-501, followed by radical polymerization of NIPAAm and methylene bis-acrylamide (BIS) (Fig. 2A). The feasibility of the TDM column was investigated using phenytoin, lamotrigine, carbamazepine, disopyramide, quinidine, propafenone, digoxin, vancomycin, mycophenolic acid, and methotrexate as TDM drugs. The elution behavior of each type of drugs containing serum as a contaminant was observed. In the chromatogram, all serum proteins and drugs were separated and eluted as two peaks with short analysis times (Fig. 2C). Serum was immediately eluted, and drug was slightly retained on the column because the serum proteins did not effectively interact with PNIPAAm and most of the drugs interacted with PNIPAAm through hydrophobic interactions. These results indicate that TDM can be performed using a PNIPAAm-modified bead-packed column with all aqueous mobile phases.

**Fig. 3:**
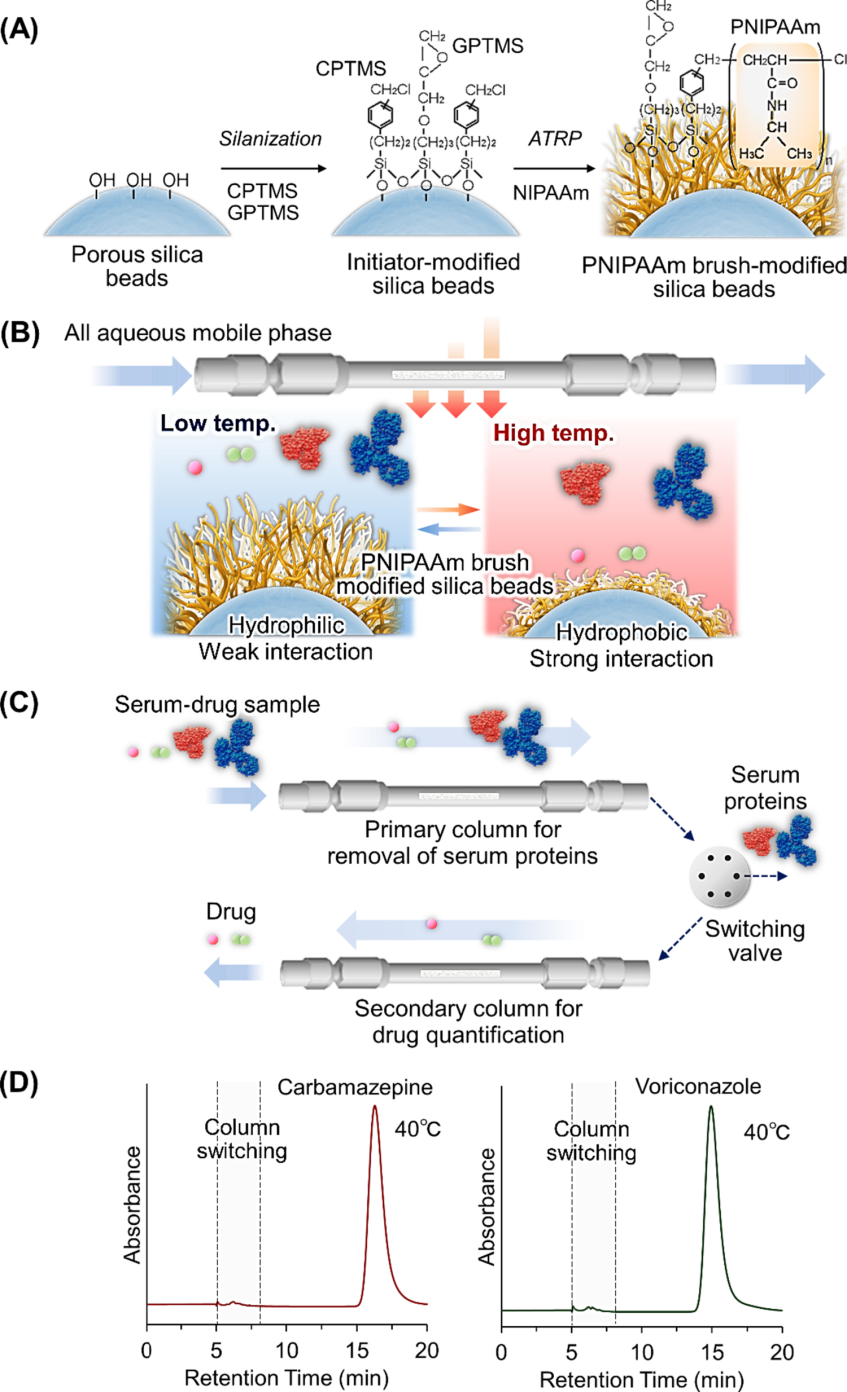
Two-dimensional temperature-responsive chromatography for TDM. (**A**) Preparation scheme for dilute PNIPAAm brush-modified silica beads, (**B**) Temperature-modulated interaction between PNIPAAm and analytes, (**C**) Conceptual diagram of two-dimensional temperature-responsive chromatography for TDM, and (**D**) Chromatograms of the drug-serum samples with column switching

Serum proteins in drug sample often adsorb the stationary phase of HPLC columns leading to the reduced function of the column and reproducibility of the drug concentration measurement. To resolve the problem, temperature-responsive chromatography was applied to a two-dimensional HPLC system (Fig. 3) [112]. Primary column was used to separate the serum protein and drug, and serum protein was flowed out and drug was introduced to the second column. The secondary column was used to determine the drug concentration.

Dilute PNIPAAm brush-modified silica beads were prepared using atom transfer radical polymerization (Fig. 3A). The density of PNIPAAm was reduced by introducing 3-glycidyloxypropyltrimethoxysilane (GPTMS) during ATRP initiator modification of the silica beads. The diluted density of PNIPAAm on the silica beads shortens the retention time of the analyte compared to the dense PNIPAAm brush, which is attributed to reduced hydrophobic interactions between PNIPAAm and the analyte [26]. The prepared columns were used in a two-dimensional HPLC system (Fig. 3C). The primary column was used to separate the serum proteins and drugs. After elution of the serum protein, the drug was introduced into the second column, and the drug concentration was determined. The elution behaviors of 13 drugs (carbamazepine, lamotrigine, zonisamide, phenobarbital, nitrazepam, diazepam, disopyramide, quinidine, propafenone, sotalol, voriconazole, lidocaine, and theophylline) were observed using two-dimensional temperature-responsive chromatography. Using a two-dimensional temperature-responsive chromatography system, drug peaks without serum peaks were observed in the secondary column (Fig. 3D). The quantitative determination of drugs in serum proteins can be performed using the chromatogram of the secondary column. The results indicated that the two-dimensional temperature-responsive chromatography system could remove serum proteins from the drug through the primary column, and precise quantitative determination was performed using a secondary column.

## TDM using temperature-responsive ion exchange chromatography

As various types of drugs have ionic properties, ion-exchange chromatography is suitable for their effective retention. Thus, temperature-responsive ion-exchange chromatography was investigated for TDM [113, 114].

**Fig. 4:**
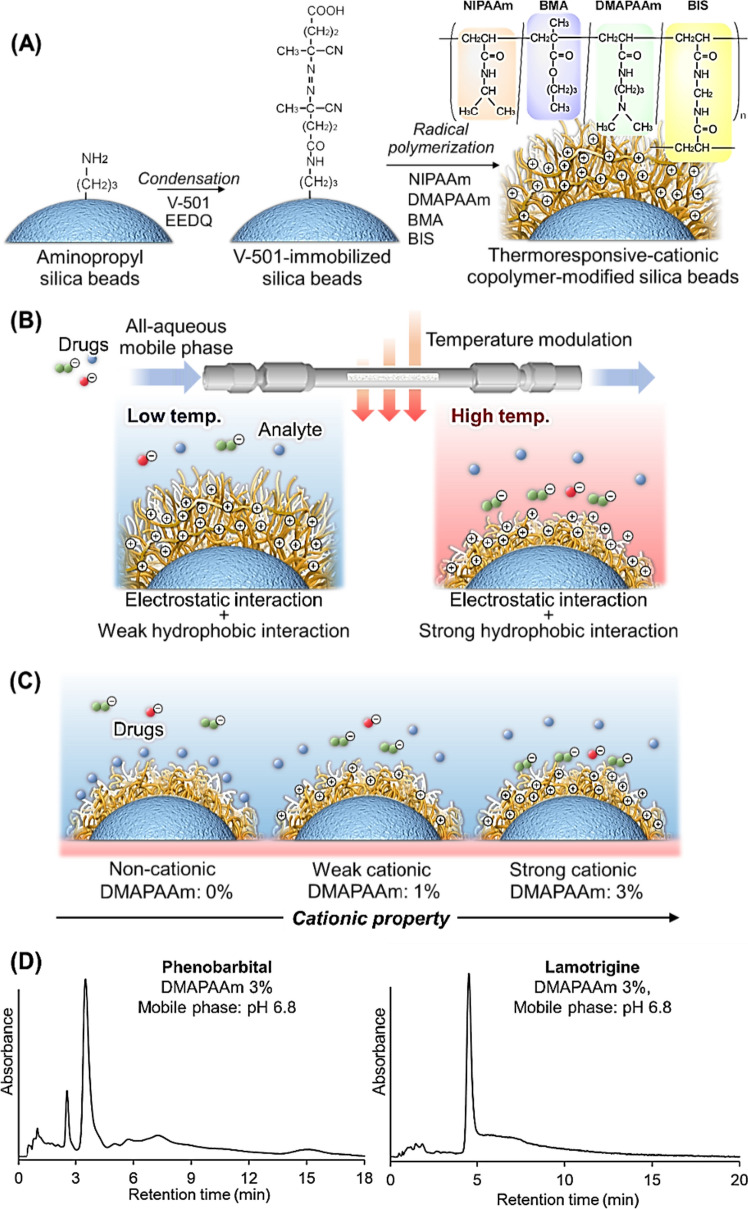
Temperature-responsive ion-exchange chromatography for TDM. (**A**) Preparation scheme for cationic copolymer-modified silica beads, (**B**) Temperature-modulated interactions between copolymer and analytes, (**C**) Conceptual diagram of cationic property of the polymer-modified silica beads, and (**D**) Chromatograms of drug sample on the prepared column

Temperature-responsive anion-exchange chromatography was developed to effectively interact with anionic drugs (Fig. 4) [113].

The cationic monomer *N*,*N*-dimethylaminopropyl acrylamide (DMAPAAm), was introduced into PNIPAAm on silica beads via copolymerization (Fig. 4A). *n-*Butylmethacrylate (BMA) was introduced to the polymer to modulate its hydrophobicity. The cationic properties of the polymers were modulated by varying the amount of DMAPAAm added. The 13 drugs, disopyramide, voriconazole, lidocaine, zonisamide, mexiletine, carbamazepine, quinidine, phenytoin, lamotrigine, diazepam, phenobarbital, sotalol, and mycophenolic acid, were used as model analytes, and the elution behavior of the drugs from the copolymer modified beads packed column was observed. Phenobarbital and lamotrigine were effectively retained on the strongly cationic polymer-modified bead-packed column (DMAPAAm3%) (Fig. 4D). Sotalol, quinidine, and mycophenolic acid effectively retained the weakly cationic polymer-modified bead-packed column (DMAPAAm1%). Voriconazole, zonisamide, diazepam, phenytoin, carbamazepine, mexiletine, lidocaine, and disopyramide were effectively retained on a noncationic polymer bead-packed column (DMAPAAm0%). This is attributed to the suitable balance between the hydrophobic and cationic interactions between the polymer and drugs. With an increase in the amount of cationic monomers in the polymer, the cationic properties improved. By contrast, with an increase in the amount of cationic monomers in the polymer, the hydrophobicity of the polymer decreased because the cationic groups provided hydrophilicity to the polymer. Therefore, polymer-modified beads with appropriate cationic and hydrophobic properties should be used to analyze each drug.

**Fig. 5:**
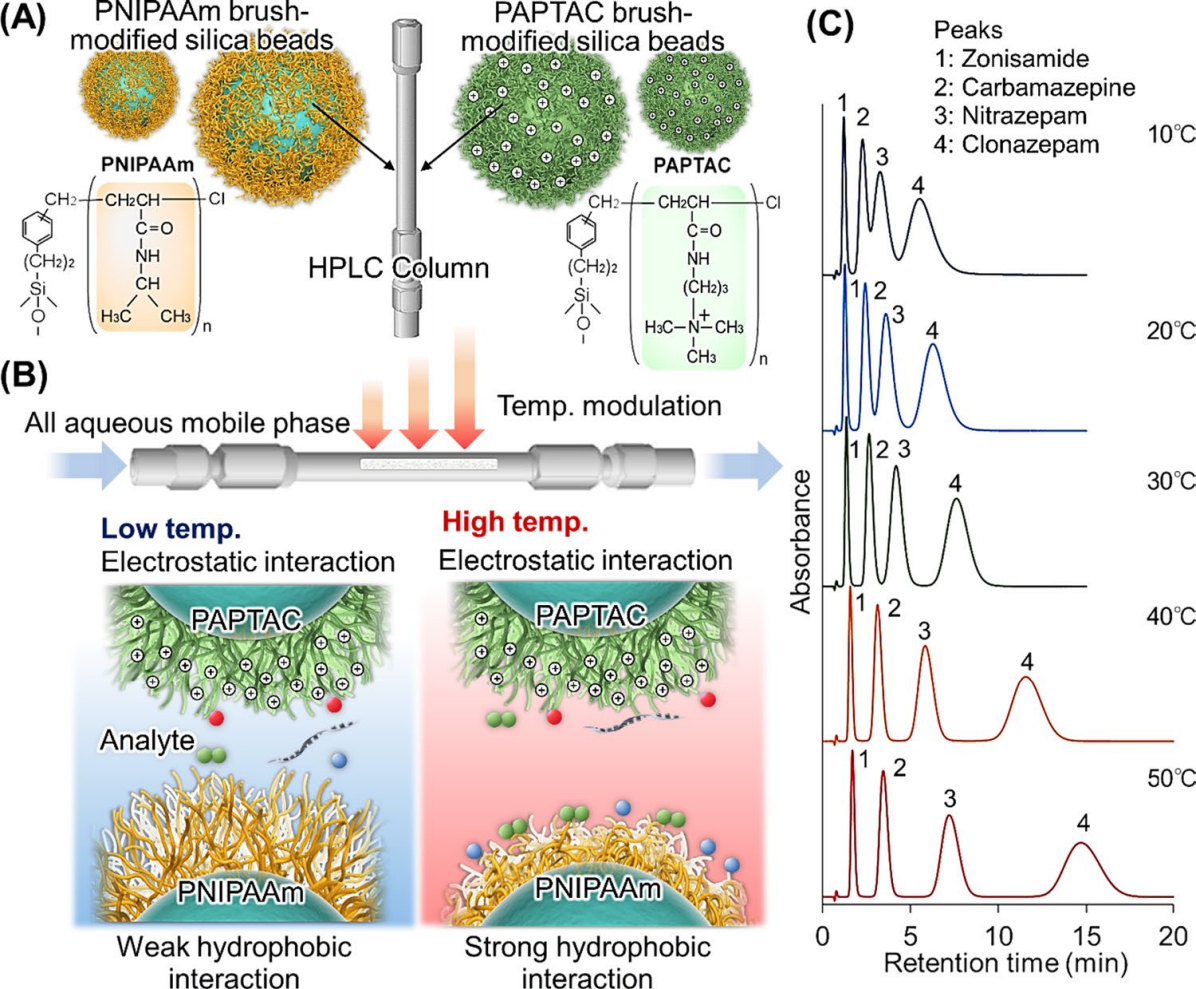
Temperature-responsive mixed-mode chromatography for TDM. (**A**) Conceptual diagram of the temperature-responsive mixed-mode column using thermoresponsive polymer-modified silica beads and cationic polymer-modified silica beads, (**B**) Temperature-modulated interactions between polymers and analytes, and (**C**) Chromatograms of antiepileptic drugs on the temperature-responsive mixed-mode column at various temperatures

Temperature-responsive mixed-mode chromatography using mixed beads effectively exploits electrostatic interactions (Fig. 5) [114, 115]. Mixed-mode columns using PNIPAAm-modified beads and poly(3-acrylamidopropyltrimethylammonium chloride) (PAPTAC) modified beads have been investigated for temperature-modulated interactions with antiepileptic drugs, which often require TDM [114]. In the mixed mode column, PNIPAAm modified beads were used for temperature-modulated hydrophobic interaction with analytes. In addition, PAPTAC modified beads were used for electrostatic interaction with analytes because PAPTAC has strong cationic property [64, 77, 116]. Using a temperature-responsive mixed-mode column, the antiepileptic drugs phenytoin, clonazepam, nitrazepam, carbamazepine, lamotrigine, phenobarbital, zonisamide, and ethosuximide are retained in the column through electrostatic and hydrophobic interactions. The retention time of these antiepileptic drugs increased with increasing temperature because of the increased hydrophobic interactions with the drugs at higher temperatures in addition to the electrostatic interactions. Additionally, mixtures of zonisamide, carbamazepine, nitrazepam, and clonazepam were separated using a mixed-mode column (Fig. 5C). The results indicate that the prepared temperature-responsive mixed-mode column can be utilized for the TDM of multidrug doses of various antiepileptic drugs.

## Sample preparation using temperature-responsive spin column

**Fig. 6:**
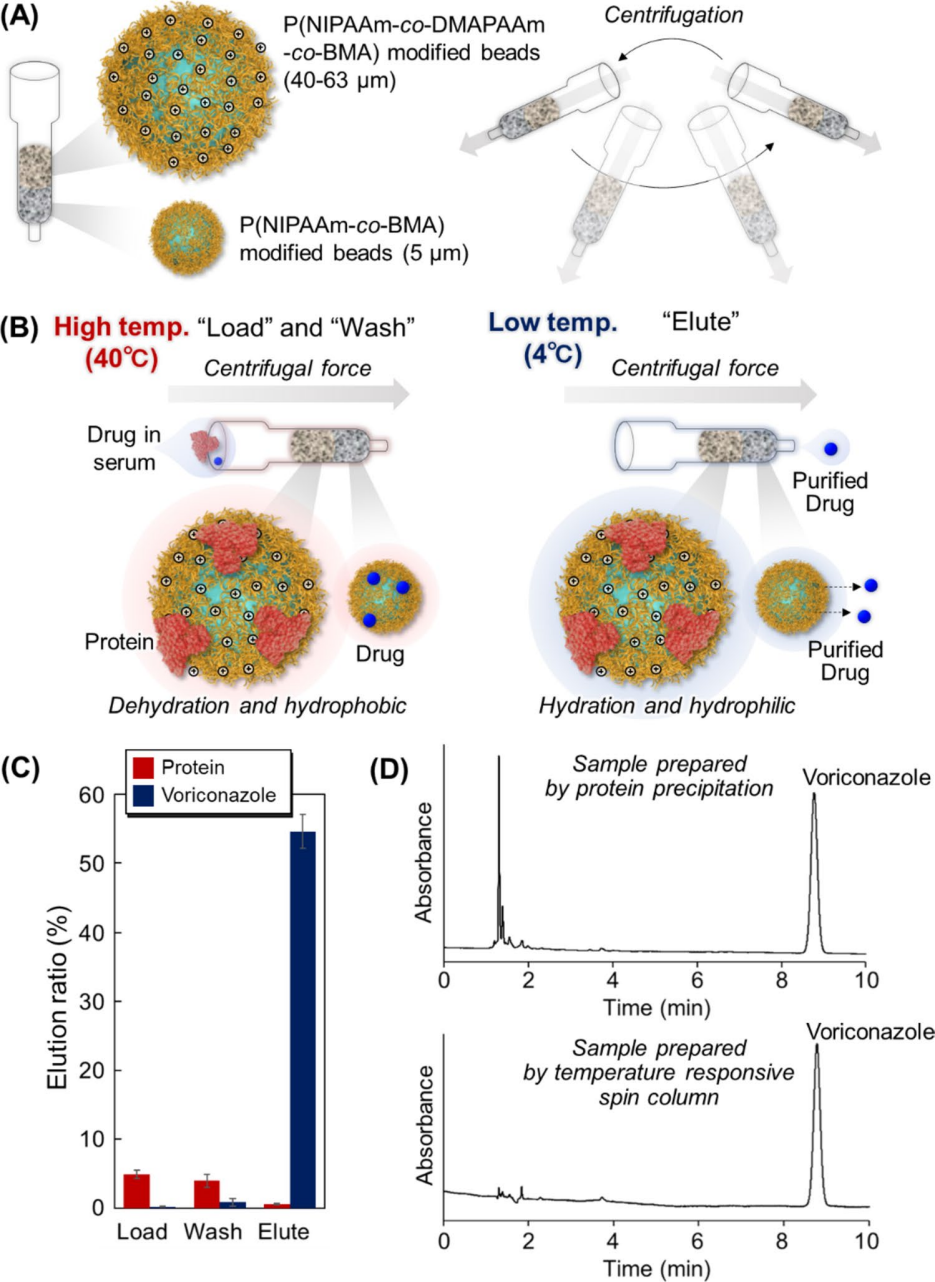
Temperature-responsive spin column for the preparation of drug-serum samples. (**A**) Temperature-responsive spin columns composed of two types of thermoresponsive beads, (**B**) Conceptual diagram of the temperature-responsive spin column, (**C**) Elution of serum proteins and voriconazole, and (**D**) Chromatograms of voriconazole and serum proteins prepared using protein precipitation and temperature-responsive spin column (adapted from [118] with permission from Elsevier)

In most cases of TDM using chromatography, sample preparation is required to remove serum proteins [117]. Various sample preparation methods, such as organic protein precipitation, solid extraction columns, and spin columns, require organic solvents that are not desirable for use in hospitals. Therefore, a sample preparation method that does not require organic solvents is desired. To resolve this issue, a temperature-responsive spin column without an organic solvent was developed for sample preparation [118] (Fig. 6). Two types of beads were prepared as the packing materials for the spin column (Fig. 6A). One was P(NIPAAm-*co*-DMAPAAm-*co*-BMA)-modified silica beads for trapping serum proteins. The beads were prepared using relatively large diameter of silica beads (40–63 μm). The other comprised P(NIPAAm-*co*-BMA)-modified silica beads for drug adsorption. The beads were prepared using relatively small diameter of silica beads (5 μm). The prepared two types of beads were packed into the spin columns as two layers; the prepared P(NIPAAm-*co*-BMA) beads and P(NIPAAm-*co*-DMAPAAm-*co*-BMA) beads were the bottom and upper layers of the spin column. Using the prepared spin column, the sample preparation of serum-voriconazole sample was performed (Fig. 6C). At 40 °C, the sample of voriconazole with serum protein was introduced to the spin column, and the spin column was centrifuged. The serum protein and voriconazole were adsorbed on the spin column at 40 °C. By reducing the temperature of the spin column and introducing cooling water into the column, the adsorbed drug was eluted from the column, whereas the protein adsorbed on the beads remained on the column. The efficacy of the spin column sample preparation was investigated by comparing protein precipitation using organic solvents (Fig. 6D). The voriconazole-serum protein samples were treated using a temperature-responsive spin column, followed by chromatographic analysis using an ODS column. Only a voriconazole peak was observed in the chromatogram after sample preparation using the spin column. By contrast, a contaminant peak was observed in the chromatogram after sample preparation via protein precipitation. The results indicate that the developed temperature-responsive spin column would be useful for sample preparation in healthcare facilities without organic solvents.

## Antibody drug separation using thermoresponsive polymer

Biopharmaceuticals have become effective medical treatments for intractable diseases [119]. Antibody drugs are promising biopharmaceutics because of their specificity for target molecules and relatively few side effects compared to low-molecular-weight chemical drugs [119–121]. The importance of separation and analysis techniques for antibody drugs has increased significantly. Numerous separation and analytical techniques have been developed for this purpose. Affinity chromatography, which employs a protein A ligand, is a powerful tool for this purpose because of the strong affinity between protein A and antibodies. Antibody elution from the columns is typically accomplished using low-pH aqueous solutions. The elution process in the antibody-drug separation column may result in the loss of activity of the antibody drugs and contamination owing to the elution of protein A. Therefore, there is a need for an antibody-drug separation column that employs distinct retention and elution mechanisms other than protein A.

Temperature-responsive chromatography using PNIPAAm is a potential technique for separating antibodies because of the ability to adjust analyte retention and elution by altering the temperature of the chromatographic system.

**Fig. 7:**
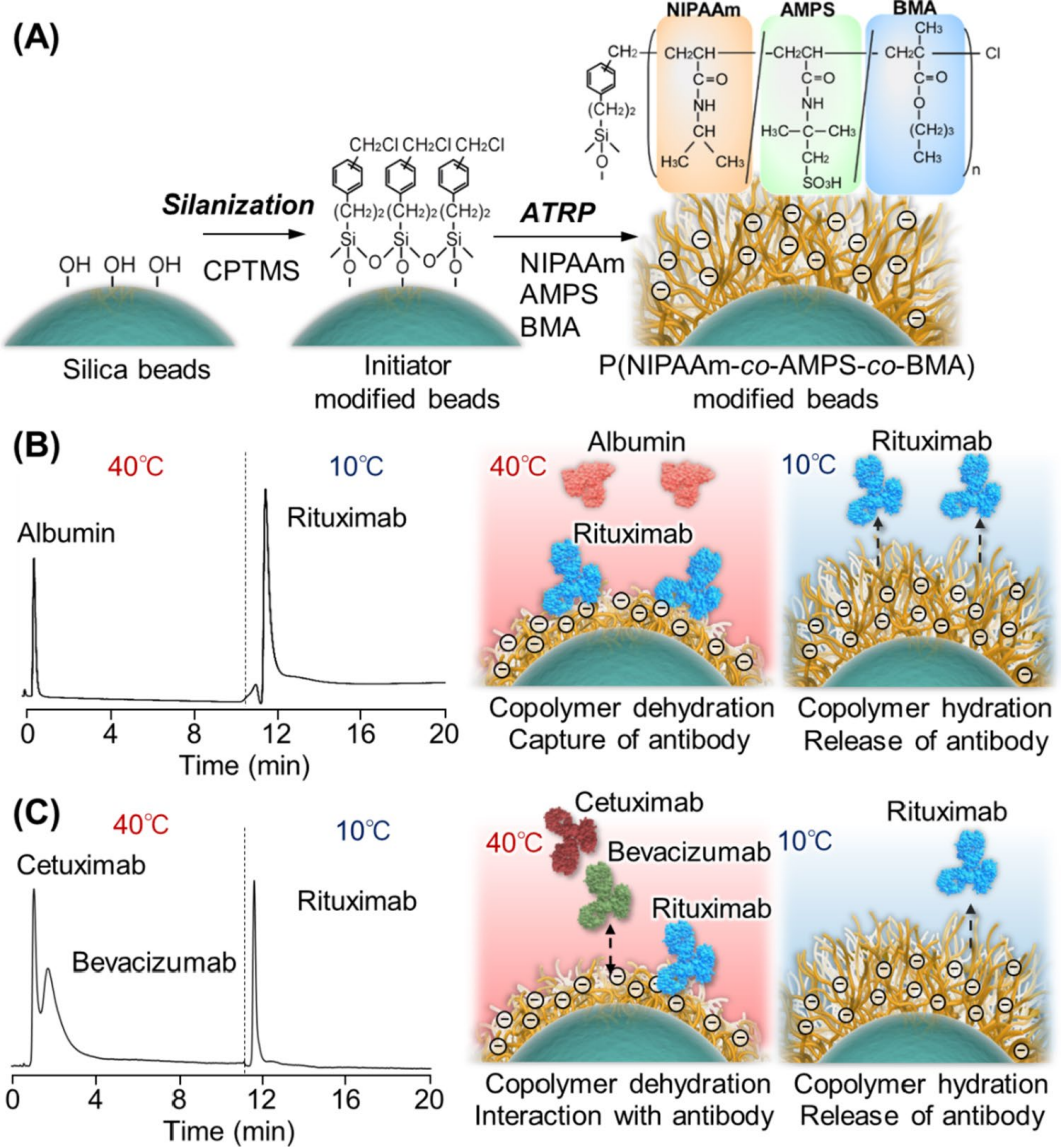
Temperature-responsive chromatography using thermoresponsive anionic polymer-grafted silica beads for antibody drug purification. (**A**) Preparation scheme for thermoresponsive anionic polymer-grafted silica beads, (**B**) Temperature-modulated purification of rituximab from albumin contaminants, and (**C**) Separation of the three antibody drugs according to temperature using the prepared column

A temperature-responsive chromatography column for antibody-drug separation was developed using PNIPAAm containing a sulfonic acid group [66] (Fig. 7). Poly(NIPAAm-*co*-2-acrylamido-2-methylpropanesulfonic acid (AMPS)-*co*-BMA)-modified silica beads were prepared by atom transfer radical polymerization (ATRP) (Fig. 7A). Using the rituximab elution profiles, the prepared bead-packed column was investigated as an antibody purification column. Rituximab is adsorbed onto the copolymer brush at high temperatures through hydrophobic and electrostatic interactions because the copolymer dehydrates and becomes hydrophobic, and the anionic properties of the AMPS are maintained. By contrast, rituximab was not adsorbed onto the copolymer brush at low temperatures because it was extended and hydrated. The purification of rituximab from contaminants, albumin or hybridoma culture media, was achieved by simply changing the temperature of the column from 40 to 10 °C (Fig. 7B). Furthermore, three types of antibody-drug mixtures, rituximab, cetuximab, and bevacizumab, were separated using a column through temperature-modulated hydrophobic and electrostatic interactions (Fig. 7C). These findings indicate that a temperature-responsive column can be employed to separate and analyze biopharmaceuticals by simply controlling the column temperature.

**Fig. 8:**
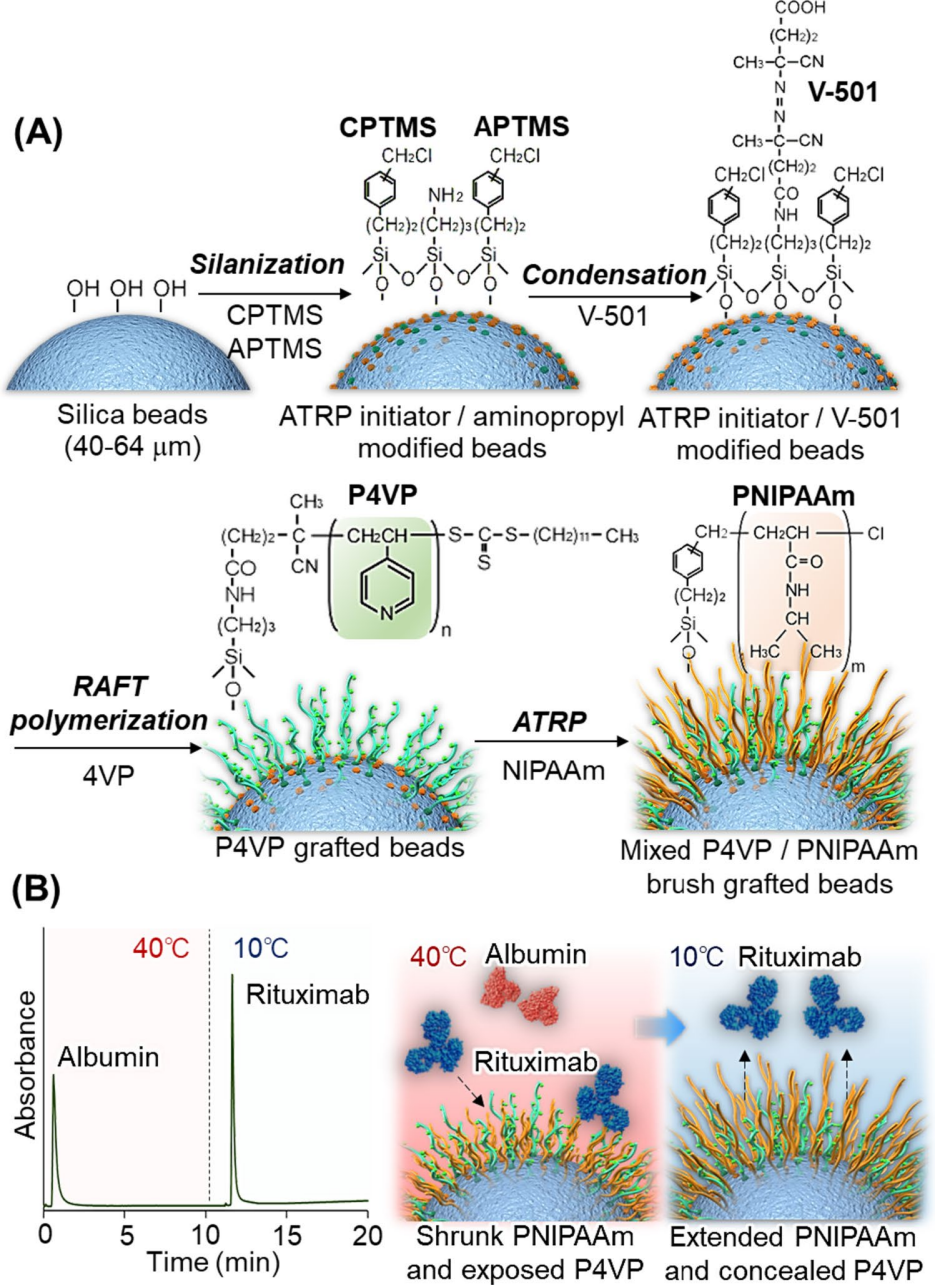
Temperature-responsive chromatography using a thermoresponsive mixed polymer brush for antibody drug purification. (**A**) Preparation scheme of thermoresponsive mixed-polymer brush-grafted silica beads and (**B**) Temperature-modulated purification of rituximab from albumin contaminants (adapted from [68] with permission from Elsevier)

Another type of antibody–drug purification column were developed by using a mixed polymer brush composed of PNIPAAm and poly(4-vinyl pyridine)(P4VP) [68] (Fig. 8). P4VP has been utilized as a ligand for protein purification columns [122, 123]. Thus, mixed PNIPAAm and P4VP brush-modified silica beads were prepared as packing materials for effective antibody–drug purification (Fig. 8). A thermoresponsive mixed polymer brush was prepared by reversible addition-fragmentation chain transfer (RAFT) polymerization of 4VP and subsequent ATRP of NIPAAm (Fig. 8A). The elution behavior of rituximab was observed at various temperatures using a bead-packed column. At high temperatures, rituximab was adsorbed on the mixed polymer brush because PNIPAAm shrunk and P4VP was exposed, leading to an increased interaction between rituximab and P4VP (Fig. 8B). By contrast, at low temperatures, rituximab did not adsorb onto the column because PNIPAAm was extended, and P4VP was concealed, preventing the interaction between rituximab and P4VP. Rituximab was purified from the contaminant using a mixed polymer brush (Fig. 8B). A mixture of rituximab and albumin was introduced to the bead-packed column at 40 °C; rituximab was adsorbed on the mixed polymer brush, whereas albumin was not adsorbed and eluted from the column. The adsorbed rituximab was eluted from the column by reducing the temperature from 40 to 10 °C (Fig. 8B).

## Conclusions

This review summarizes recently developed temperature-responsive chromatography systems for clinical applications and their properties. The PNIPAAm-modified silica bead-packed column effectively separated drugs and serum proteins, allowing the determination of the quantity of drugs in the serum sample. In addition, quantitative analysis of drugs in serum samples was performed using a thermoresponsive cationic copolymer-modified bead-packed column. The antiepileptic drugs were separated using a temperature-responsive mixed-mode chromatography column. Serum sample preparation without organic solvent was performed using the developed temperature-responsive spin column. Antibody drugs were purified from contaminants using the developed temperature-responsive chromatography column by simply changing the temperature. Therefore, the developed PNIPAAm-based temperature-responsive chromatography system has the potential to function as an effective separation and analytical method for various types of medicines, including small-molecule drugs and biopharmaceuticals. These temperature-responsive chromatography systems can also be utilized for clinical diagnosis, as they can assess multiple medicines simultaneously. This highlights the significant potential of temperature-responsive chromatography in medicine and healthcare.

## Data Availability

The data used in the current study are available from the corresponding author upon reasonable request.
